# On the Path to UHC – Global Evidence Must Go Local to Be Useful: Comment on "Disease Control Priorities Third Edition Is Published: A Theory of Change Is Needed for Translating Evidence to Health Policy"

**DOI:** 10.15171/ijhpm.2018.118

**Published:** 2018-12-10

**Authors:** Austen Davis, Damian G. Walker

**Affiliations:** ^1^Norwegian Agency for Development Cooperation (Norad), Oslo, Norway.; ^2^Bill & Melinda Gates Foundation, Seattle, WA, USA.

**Keywords:** Health Sector Reform, Technical Assistance, Institutional Capacity Building, Health Financing, Universal Health Coverage

## Abstract

The Disease Control Priorities (DCP) publications have pioneered new ways of thinking about investing in health. We agree with Norheim, that a useful first step to advance efforts to translate DCP’s global evidence into local health priorities, is to develop a clear Theory of Change (ToC). However, a ToC that aims to define how global evidence (DCP and others) can be used to inform national policy is too narrow an undertaking. We propose efforts should be directed towards developing a ToC to define how to support progressive institutional development to deliver on universal health coverage (UHC), putting the client at the center. Enhancing efforts to meet the new global health imperatives requires a shift in focus of attention to move radically from global to local. In order to achieve this we need to reorganize the nature of technical assistance (TA) along three major lines (1) examine and act to clarify the mandates and roles to be played by multilateral normative and convening agencies, (2) ensure detailed understanding of local institutions, their needs and their demands, and (3) provide TA over time and in trust with local counterparts. This last requirement implies the need for long-term local presence as well as an international network of expertise centers, to share scarce technical capabilities as well as to learn together across country engagements. Financing will need to be reorganized to incentivize and support demand-led capacity strengthening.

The Disease Control Priorities (DCP) publications^1-3^ have pioneered new ways of thinking about investing in health (economic benefits of good health as well as the importance of making choices in what to invest in). The third edition has adapted to critiques that it was too narrowly focused on cost-effectiveness and has incorporated additional considerations – it has broadened the range of health concerns to be evaluated – and it has provided policy advice on options for assembling essential health benefits packages for resource-constrained settings. However, to date, while editions 1 and 2 had some impact at the global level, examples of use among national policy-makers have been limited.^4^ Along with the global publication of DCP3, focused efforts have been put in place to translate contents to country-level uses in Afghanistan and Ethiopia^ [1] ^. Clearly, additional efforts are required to translate the rich evidence included in DCP into national policy, to ensure global evidence has vital local meaning.^5^

We agree with Norheim,^6^ that a useful first step to advance efforts to translate DCP’s global evidence into local health priorities is to develop a clear Theory of Change (ToC) that articulates the desired long-term country outcomes. We also concur with Norheim’s detailed assumptions about the need for local capacity and institutions as requirements for success. Where we differ from Norheim is in emphasis. If the aim is for long-term country outcomes (better governance of health systems for efficiency and equity) then we need a ToC that emphasizes the primary process – development of public capacity. While the use of excellent global knowledge is of great importance, it is a source of knowledge as opposed to the process of change we are interested in.

Once we put the client at the center, a broader range of critical assumptions and intervention issues become explicit – and the pathways to the outcomes we are looking for (better governance of health systems for efficiency and equity) are made more realistic. In this commentary we try and broaden the frame for discussion by arguing that efforts to integrate global evidence into priority-setting need to be seen within a broader context of supporting local capacity to undertake a range of functions to realize health financing reform.

The changing demography, burden of disease and domestic and international agenda for health mean that countries need much more complex health systems and a wider range of capacities to govern them. Countries must plan, within fiscal space constraints, to diagnose and treat a far wider range of conditions for a broader segment of the population. They must also financially protect health care users; regulate and partner with private sector providers; protect the public from relevant risk factors (such as poor diet, air pollution, misuse of alcohol, and road traffic accidents); and build capacity to detect and respond to outbreaks of potentially epidemic diseases.

As opposed to having processes led by those intent on transferring a body of global knowledge or a specific set of technologies (eg, health technology assessment or strategic purchasing), it might be better to partner with national governments to strengthen or build institutions by asking first “what are the existing capabilities?” and “what is needed?” The desired outcome must be locally sustainable, multi-functional, learning institutions – able to govern national health systems effectively, and within existing resource constraints. In short, we believe it may be more effective to develop a ToC to support progressive institutional development to deliver on universal health coverage (UHC). This puts the client at the center, as opposed to the knowledge provider. But how to get there?

Global knowledge can only be used by countries if there are the institutions, processes and capacities to be able to translate it into a series of decisions – implement – and reiterate those decisions dynamically over time. This is why thinking deeply about the role of evidence at country-level – how to support institutions to make decisions and implement these decisions is critical. This involves a reimagining of the role of national governments in resource-constrained settings to have a broader and more complex function. Specifically, this means governments must have capabilities in priority-setting (the focus of DCP), but also health financing, equity analysis, private sector regulation and strategic purchasing, information systems for evidence driven policy-making, and accountability and inclusion of a wide range of societal interests.^7^ The global agenda goes from driving towards globally established targets (eg, reduction of under 5 mortality or HIV transmission rates) to national development of systems, and becomes domestic and political.

Our proposed conceptualization should consider how development assistance for health (DAH) can better support national governments to emerge as competent, dynamic, sovereign agencies for health promotion and protection while pursuing results.

Technical assistance (TA) has long been a major component of aid – reflecting a desire to transfer technology/knowledge and not just financial resources. But TA is often provided piecemeal, with short-term perspectives and across a wide and uncoordinated range of issues that governments have not necessarily prioritized, often occurring without clear strategies for transfer or sustainability.

(1) At the country level – the institutional arrangements need to be clear and the loci of responsibility established. Defining which national institute or unit should be supported with TA is important – this would lead to all relevant TA in one thematic area being delivered to the same unit by different agencies. This would allow a better overview, comparison of worth and coordination of TA by national governments.
(2) TA should be demand-driven as opposed to supply-driven. TA is too often driven by the mandate and or technical position of the TA providing institution (with control of TA resources). No matter how technically correct the perceived priorities for what a national government should be doing – it cannot do everything. If the national government has not prioritized a particular issue, no amount of excellent TA will drive purposeful processes leading to decisions and implementation. In order to be effective, TA should be provided to answer the questions the national government is interested to answer – and to collectively find an appropriate compromise within political and operating constraints (as opposed to upholding “global recommendations” without consideration of local factors or feasibility). This will require expertise but also real-world experience from TA providers. Countries may want to source TA from actors within the region or from other countries with recent relevant experience as opposed to Western experts.
(3) Such TA needs to be provided by a trusted partner. TA providing partners might set up long term relationships with the institution in question – responding to a variety of requests and needs over time as the institution progresses down the pathway of building capacities and defining and answering critical questions.
(4) Plans for reform of World Health Organization (WHO) country offices (under the new 13th General Programme of Work) include a reformulation of their role and competence in-country.^8^ The WHO should focus less on providing TA and more on helping to identify critical needs/priorities, the unit that is the loci of responsibility and potential partners to provide TA (ie, work on the demand-side not supply-side as well). Under such operating conditions WHO would then have greater legitimacy to convene networks of providers involved in such long term support processes – and allow them to exchange expertise, staff and learning (to enhance their TA support value) - and concretize this learning as part of WHO’s normative processes.
(5) Much TA and capacity support is provided in the form of training and or advice to individuals. Too little is considered in terms of building institutions. In addition to specific technologies – TA providers need to look at the legal mandate of institutions – administrative processes – board oversight – technical resources and staffing issues.

It is critical that national governments can source useful and appropriate TA for what is needed and demanded over time. There is some good news:

- Development partners, including international organizations, bilateral, regional and multilateral banks, foundations and others, are increasingly offering support to strengthen national institutional capacity for evidence-informed decision-making for UHC. This includes defining strategic support functions for UHC and building global knowledge^ [2] ^.
- Bilateral and multilateral donors are united in their awareness that they can no longer only focus on achieving global health outcomes because of unintended impacts on broader health system performance. The Global Financing Facility in support of Every Women Every Child (GFF) is a relatively new initiative that aims to do business differently – explicitly leveraging grant investments to elicit government leadership, strategic planning and domestic resource mobilization. The GFF shows considerable promise.^9^
- The US Agency for International Development (USAID) has recently selected Results for Development to lead a new 5-year global initiative: The Health Systems Strengthening Accelerator (HSS Accelerator). This global platform will “connect locally-driven health system reforms and innovations with global knowledge, enhance local institutions devoted to ongoing system strengthening, and accelerate countries’ journeys to self-reliant health systems.”^10^

Donors realize that DAH must not cause harm and if possible must realize meaningful benefits for the health of ordinary people, whilst propelling health system development forward. The initiatives listed above are a step in the right direction, but they remain fragmented and uncoordinated. The “whole of DAH” impact at country level is not sufficiently considered and no one takes responsibility for it. New initiatives are promising and indicative of increased will to grapple with these issues. However, regarding support to the emergence of competent national health systems, able to drive programmes of reform over time, much still needs to be done.

The shifts in strategically connecting global knowledge, the role of global convening agencies and new approaches to the provision of TA, that we are proposing, is not happening sufficiently. What is sorely needed is a coordinated approach and platform with a shared ToC for TA for institutional capacity building to support health sector reform. Some of this thinking has already been outlined in the Bangkok Statement: Priority Setting for Universal Health Coverage.^11^

While there are undoubtedly problems with how TA for priority-setting and health systems support more generally is currently offered, an opportunity clearly exists. There is an urgent need to work together to create joined up aid investments to ensure progress on health and domestic capacity at the same time – and across a range of health concerns. We think further work is needed to elucidate the role of global knowledge, global institutions (such as WHO and the World Bank) and centers of expertise (TA providers). We also believe redesign must pivot around national institutional imperatives and not global health interests. TA – and the interests of TA providers – should be aligned with national interests.

Governments of resource-constrained countries, global health financiers and experienced knowledge partners must work better together to define partnerships of genuine use to national governments, and to network them to learn collectively and expand impact across country borders.

We believe there is a role for DCP’s global evidence, and that through translation efforts at country level, DCP can play an important role in effecting resource allocation decisions for health, and thereby produce more health for the money. But for this outcome to become a reality, it will require broader coordination efforts by a range of partners, including WHO, the World Bank and GFF, other Global Funds (GAVI and GFATM) and USAID’s HSS Accelerator, to define roles and to ensure emphasis on coordination and local ownership.

## Acknowledgements

The perspectives raised are those of the authors alone and should not be taken as indicating the policy position of their affiliated agencies (Norad and BMGF).

## Ethical issues

Not applicable.

## Competing interests

Both authors work for donor agencies financing DCP (the subject of the primary article) and other initiatives named in the article.

## Authors’ contribution

Both authors worked on drafting the statement following a request by the Journal for a commentary.

## Endnotes


[1] For Ethiopia see: https://www.uib.no/en/rg/globpri/110822/gates-funds-ethiopian-health-priorities-project-3-million.

For Afghanistan see: https://www.lshtm.ac.uk/newsevents/expert-opinion/developing-new-basic-package-health-services-afghanistan-0.

[2] For example DCP, the International Decision Support Initiative (iDSI), the Strategic Purchasing Africa Resource Center (SPARC), the Joint Learning Network (JLN), the Primary Health Care Performance Initiative (PHCPI), to name a few.


## Authors’ affiliations

^1^Norwegian Agency for Development Cooperation (Norad), Oslo, Norway. ^2^Bill & Melinda Gates Foundation, Seattle, WA, USA.
